# Prediction of Possible Biomarkers and Novel Pathways Conferring Risk to Post-Traumatic Stress Disorder

**DOI:** 10.1371/journal.pone.0168404

**Published:** 2016-12-20

**Authors:** Kumaraswamy Naidu Chitrala, Prakash Nagarkatti, Mitzi Nagarkatti

**Affiliations:** Department of Pathology, Microbiology and Immunology, School of Medicine, University of South Carolina, Columbia, South Carolina, United States of America; University of Nottingham, UNITED KINGDOM

## Abstract

Post-traumatic stress disorder is one of the common mental ailments that is triggered by exposure to traumatic events. Till date, the molecular factors conferring risk to the development of PTSD is not well understood. In this study, we have conducted a meta-analysis followed by hierarchical clustering and functional enrichment, to uncover the potential molecular networks and critical genes which play an important role in PTSD. Two datasets of expression profiles from Peripheral Blood Mononuclear Cells from 62 control samples and 63 PTSD samples were included in our study. In PTSD samples of GSE860 dataset, we identified 26 genes informative when compared with Post-deploy PTSD condition and 58 genes informative when compared with Pre-deploy and Post-deploy PTSD of GSE63878 dataset. We conducted the meta-analysis using Fisher, roP, Stouffer, AW, SR, PR and RP methods in MetaDE package. Results from the rOP method of MetaDE package showed that among these genes, the following showed significant changes including, *OR2B6*, *SOX21*, *MOBP*, *IL15*, *PTPRK*, *PPBPP2* and *SEC14L5*. Gene ontology analysis revealed enrichment of these significant PTSD-related genes for cell proliferation, DNA damage and repair (p-value ≤ 0.05). Furthermore, interaction network analysis was performed on these 7 significant genes. This analysis revealed highly connected functional interaction networks with two candidate genes, *IL15* and *SEC14L5* highly enriched in networks. Overall, from these results, we concluded that these genes can be recommended as some of the potential targets for PTSD.

## Introduction

Posttraumatic stress disorder (PTSD) is a psychiatric condition that occurs in response to a severe traumatic event [1]. Epidemiological studies show that nations such as South Africa have 73.8%, Europe and Japan 54–64%, Spain 54%, Italy 56.1% and Northern Ireland has 60.6% lifetime traumatic event prevalence rate [2]. PTSD prevalence rate amounts to 8% with a higher rate among the people living in high-violence areas and in combat veterans [3–5]. A lifetime risk of an adult American is estimated to be 6.8% with a conditional risk ranging from 5–31% [6].

Some of the symptoms associated with PTSD include long-lasting psychological suffering, distressing psychosocial disability, reduced health-related quality of life and increased morbidity and mortality [7]. Clinical and demographic factors that increase the risk of PTSD include severity, duration of trauma, peri-traumatic dissociation, childhood abuse and lack of social support [8].

Some of the other factors conferring risk to PTSD include heritability [9–12], family instability [13], biological factors [14], endocrine factors [15], neurochemical factors [16, 17], neurocircuitry factors [18, 19], genetic factors [20–23], gender differences [24], early developmental factors [25] and physical trauma [26]. Among these, genetic factors are known to confer 30% of the variance in PTSD [27]. Several studies on the genetic and epigenetic risk factors conferring susceptibility to PTSD has been gradually increasing in the recent years [28, 29]. A recent genome-wide association study showed that single nucleotide polymorphisms within several candidate genes are known to confer significant risk to PTSD [30]. A cohort-based study showed a set of differentially expressed genes are associated with PTSD in trauma-exposed white non-Hispanic male veterans [31]. Further, several studies showed that gene-environment interactions, epigenetics and genetics of treatment response are important for PTSD susceptibility [32].

Though prediction and analysis of the genes, gene-environment interactions and genetic pathways will augment in understanding the different mechanisms of PTSD progress and recuperation, little progress has been made on identifying the candidate genes or genetic variants influencing the liability to PTSD due to methodological problems such as expediency of samples and failure in assays [33]. In order to fulfill these pitfalls in understanding the genetics of PTSD, we have performed a systematic meta-analysis to evaluate the expression of genes and pathways as predictive markers for elucidating the outcomes of PTSD.

## Materials and Methods

### Identification of gene expression datasets for PTSD

We used the NCBI GEO database (Gene Expression Omnibus) (http://www.ncbi.nlm.nih.gov/geo/) [34] for identifying the expression datasets of PTSD. We used the keyword "PTSD" for our search (Searched on 17th July 2015). Parameters such as (1) GEO accession number (2) sample type (control or PTSD) (3) platform type (Affymetrix or Agilent or Illumina or other) and (4) gene expression data were extracted from each selected dataset.

### Data normalization and quality control analysis

The selected datasets extracted from GEO database were analyzed using GeneSpring 13.0 GX software (Agilent). Raw data for all the samples in the respective datasets were summarized using the Robust Multi-Array Average (RMA) method [35]. The samples from the datasets were normalized to a threshold raw signal 1.0 using Percentile shift normalization algorithm with a percentile target of 75. To analyze the similarity between the samples representing the same experimental condition, we have performed correlation and Principal component analysis (PCA) for each experimental condition. Correlation and PCA can be performed on either entities or conditions. Since our aim is to analyze the similarity between the samples, we have selected correlation and PCA on conditions for our study. To remove false positives due to the grouping of dissimilar expression profiles, we have filtered the probe sets by expression with an upper percentile cut-off of 100 and a lower percentile cut-off of 20.

### Analysis of differentially expressed genes and hierarchical clustering

Before identifying the genes that are differentially expressed in control and PTSD samples, we subjected all control samples in the two datasets to Venn diagram analysis for understanding the specific and commonly dysregulated probes/genes (based on Entrez Gene ID) among them. Hierarchical clustering was performed to classify the analyzed samples based on gene expression profiles and to observe the overall gene expression patterns in each condition of the datasets using Genesis software [36]. The relationship between each gene across each dataset and conditions were visualized using circos plot [37].

### Meta-analysis of differentially expressed genes

To seed out most significant genes among the differentially expressed probe sets we performed meta-analysis using an in-house written R software programming code utilizing Bioconductor Gene filter package [38]. We used microarray meta-analysis method MetaDE package to analyze these probe sets. In the MetaDE package we used the methods Fisher (X^2^_Fisher_ = -2Ʃ_i = 1_ log(p_ik_) where p_ik_ represents the i^th^ gene from the k^th^ dataset following a chi-squared distribution with 2k degrees of freedom under null hypothesis) [39], roP (takes the r^th^ order among sorted *P* values of K combined studies following a beta distribution with a shape parameters r and K− r + 1 under the null hypothesis) [40], Stouffer (Z_stouffer_ = ^k^Ʃ_i = 1_ Z_i_ / √ k where z_i_ = Φ^−1^(p_ik_) and Φ represents the standard normal cumulative distribution function) [41], AW (U = —^k^Ʃ_k = 1_ W_ik_ log p_ik_ where p_ik_ represents the *P-*value of gene i in study k, and w_k_ represents the weight assigned to the k^th^ study) [42], SR (SR_i_ = ^k^П_k = 1_R_ik_ where R_ik_ represents the rank of *P* value of gene i in study k) [43], PR (PR_i_ = ^k^П_k = 1_R_ik_ where R_ik_ represents the rank of *P* value of gene i in study k) [43] and RP (RP_i_ = (^k^П_k = 1_^R^Ʃ_r = 1_pFC_irk_)^1/R^ where pFC_irk_ is the pFC value of gene i in study k under pairwise comparison r) [44, 45] for evaluation and comparison. We have filtered out the 30% of the unexpressed and non-informative genes using MetaDE.filter function. The datasets formats and the R program source codes are provided at doi: 10.6084/m9.figshare.4168014.

### Functional and biological processes enrichment analysis

Gene ontology (GO) is one of the powerful tools for the unification of biology from the genome annotations [46]. For analyzing the differentially expressed genes (DEGs) at functional and pathway level, we used DAVID (Database for Annotation, Visualization and Integrated Discovery); a web based comprehensive database of an integrated and comprehensive set of analytic tools for extracting the biological information from various genomic and proteomic studies [47]. We selected GO for gene ontology and KEGG [48], REACTOME [49], BIOCARTA, BBID [50] for pathway enrichment analysis in DAVID with a classification stringency option highest and a threshold p-value ≤ 0.05.

### Construction of interaction networks

Interaction network analysis could help in visualizing the functional links between differentially expressed genes and the other genes at the molecular level [51], which will further be useful for understanding the molecular mechanism and essentiality of genes in a particular disease. We used Ingenuity Pathway Analysis tool (IPA; Ingenuity Systems, Redwood city, CA) to show upstream regulators as well as protein downstream of each informative gene based on published, publically available data. Further, we used the Search Tool for the Retrieval of Interacting Genes (STRING) [52]; a database to provide both experimental and predicted interaction information to construct the interaction network for the genes predicted to be significant by MetaDE methods. These interactions were analyzed according to their confidence scores.

## Results

### Datasets included for our study

Search for gene expression datasets of PTSD in PBMCs showed two GEO datasets namely GSE860 [53] and GSE63878 [54] (S1 Table) containing control (normal), PTSD, Pre-deployment and Post-deployment PTSD patient samples. Subjects in the GSE860 dataset were males and females who experienced a life-threatening event meeting DSM IV [55] with an age between 16 and 65 years, ethnic origin Jewish Ashkenazi, Jewish Sephardic and Jewish mixed [53]. Subjects in GSE63878 dataset, on the other hand, were males participated in either Marine Resilience Study or Marine Resiliency Study II and prospective studies of well-characterized U.S. Marines scheduled for combat deployment to Iraq or Afghanistan with age ranging from 22 and 23 years, ethnic origin African American, Native American Mexican, Asian and other [54]. These two datasets were included in our study. The list of samples along with their respective conditions in the two datasets was provided in the S2 Table.

### Quality control analysis of the samples

**Fig 1:**
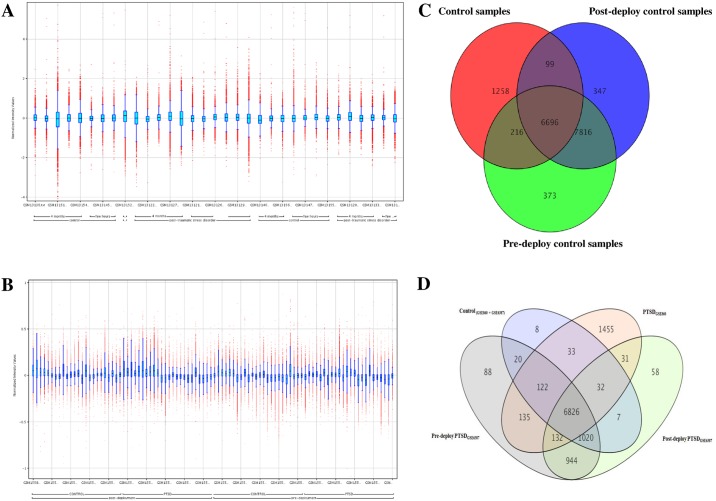
Normalization and differential expression analysis of datasets. (A,B) represents the box-whisker plot of the samples in the GSE860 and GSE63878 datasets. The middle line of each box-and-Whisker plot represents the median expression level in each sample of the dataset (C) represents the Venn diagram showing the control samples from the datasets used for our study. The result showed 6696 genes commonly modulated in control (red) and pre-(blue), post-deploy (green) control samples of both GSE860 and GSE63878 datasets (D) represents four-set Venn diagram showing the probes/genes dysregulated between samples of the datasets GSE860 and GSE63878.

All the samples from the two datasets were normalized and their respective box-whisker plots were shown in Fig 1A and 1B. Summary statistics of the dataset samples are provided in S3 Table. PCA results showed that two samples GSM13151, GSM13152 in the control condition of GSE860 dataset and two samples GSM1558862, GSM1558797 in the Post-deployment control condition and Pre-deployment PTSD condition of the GSE63878 dataset were dissimilar from rest of the samples in same experimental condition. Therefore, these four samples were excluded from the analysis. The respective correlation and PCA plots for the samples in the datasets were provided in S1 and S2 Figs. Results from filtering of probe sets (genes) by expression screened 9199 of 12600 (control samples of GSE860 dataset), 15101 of 22034 (pre-deployment control samples of GSE63878 dataset), 14958 of 22034 (post-deployment control samples of GSE63878 dataset), 8766 of 12600 (PTSD samples of GSE860 dataset), 15083 of 22034 (pre-deployment PTSD samples of GSE63878 dataset), 14589 of 22034 (post-deployment PTSD samples of GSE63878 dataset) entities from the respective samples.

### Analysis of the differentially expressed genes

**Fig 2:**
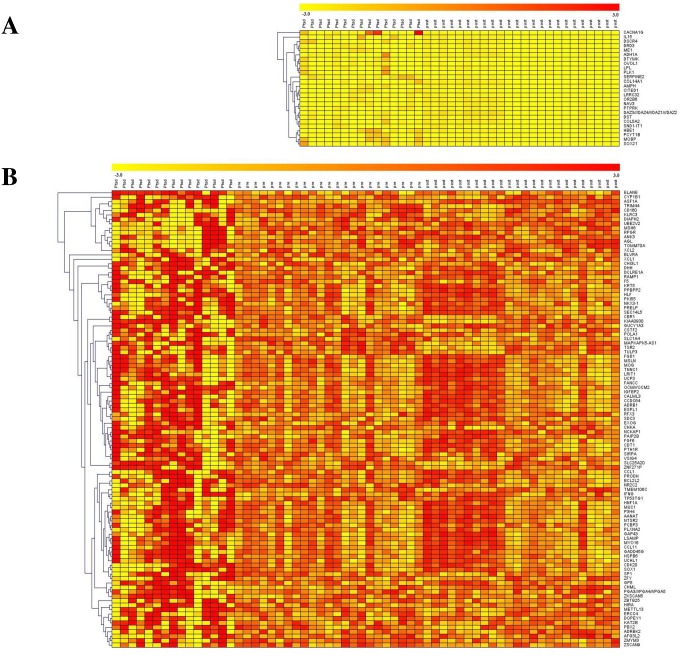
Hierarchical clustering of differential expressed genes in the datasets. (A) Represents PTSD_GSE860_ (yellow) vs Post-deploy PTSD_GSE63878_ (red) condition (B) Represents PTSD_GSE860_ (red) vs Pre-deploy PTSD_GSE63878_ (orange) vs Post-deploy PTSD_GSE63878_ (red) condition. All the probes were clustered based on normalized signal intensity ratios. Each row represented a single gene; each column represents the samples in each condition with an average linkage of expression.

Results from the Venn diagram analysis showed that 99 probes/genes were commonly dysregulated between control samples of GSE860 dataset and post-deployment control samples of GSE63878 dataset, 216 probes/genes were commonly dysregulated between control samples of GSE860 dataset and pre-deployment control samples of GSE63878 dataset, 7816 probes/genes were commonly dysregulated between pre- and post-deployment control samples of GSE63878 dataset and 6696 probes/genes were found to be commonly dysregulated among all the three control condition types (Fig 1C). Further, 1258 and 347 and 373 probes/genes were found to be specifically dysregulated for each control condition type. The 6696 probes/genes which are found to be dysregulated in all the control conditions were selected for further analysis. These 6696 probes/genes were compared with the samples of PTSD from GSE860 dataset and pre- and post-deployment PTSD samples of the GSE63878 dataset. Results showed that 132 genes were differentially expressed from the control in PTSD_GSE860_, Pre-deploy PTSD_GSE63878_ and Post-deploy PTSD_GSE63878_ samples and 31 genes were differentially expressed from the control in PTSD_GSE860_ and Post-deploy PTSD_GSE63878_ samples (Fig 1D). For the convenience, we will represent these 31 genes as PTSD_GSE860_ vs Post-deployment PTSD_GSE63878_ condition and 132 genes as PTSD_GSE860_ vs Pre-deployment PTSD_GSE63878_ vs Post-deployment PTSD_GSE63878_ condition throughout this article. Among the 132 genes that are dysregulated, 76 were found to be up-regulated and 56 genes were down-regulated with a fold change > = 1.0 and among the 31 genes that are dysregulated, 20 were found to be up-regulated and 11 genes were down-regulated with a fold change > = 1.0. The respective list of up and down-regulated genes was provided in the S4 and S5 Tables. Results from the hierarchical Euclidean cluster analysis showed distinguishable expression profiling in up and down- regulated genes between samples in each condition (Fig 2A and 2B).

### MetaDE analysis of the significantly expressed genes

**Fig 3:**
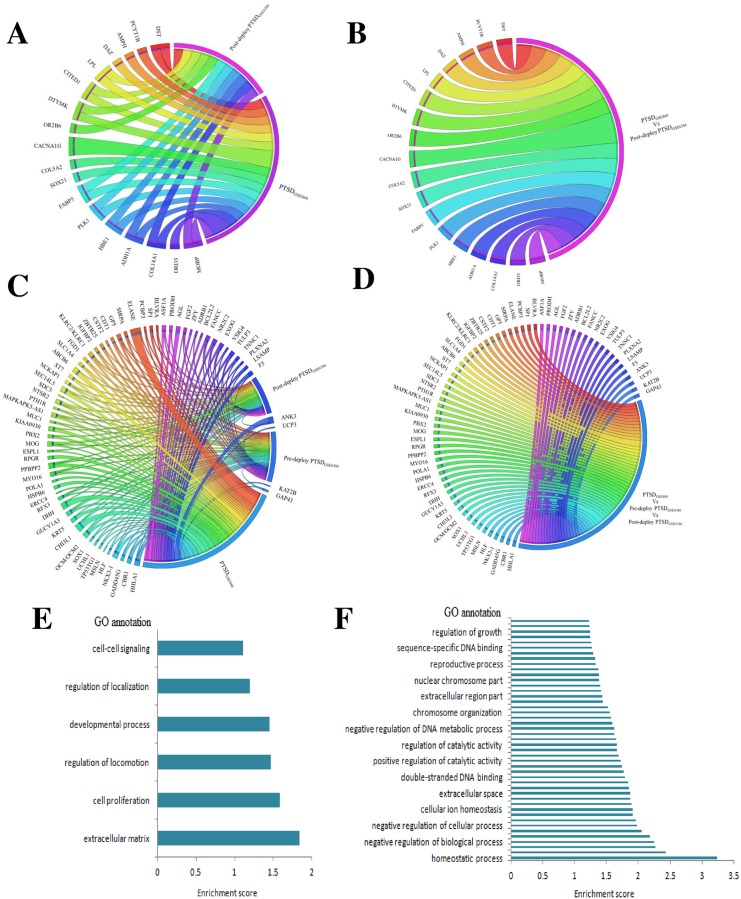
Circos plot and Gene Ontology (GO) enrichment analysis at a *P* value ≤ 0.05. (A, C) Shows the expression of the up-regulated informative genes in relation to the corresponding datasets (B, D) shows the expression of the up-regulated informative genes in PTSD_GSE860_ vs Post-deploy PTSD_GSE63878_ condition and PTSD_GSE860_ vs Pre- vs Post-deploy PTSD_GSE63878_ condition. Each connection between a gene and the condition represents the absolute fold change (E, F) Represents the gene ontology in PTSD_GSE860_ vs Post-deploy PTSD_GSE63878_ condition and PTSD_GSE860_ vs Pre- vs Post-deploy PTSD_GSE63878_ condition.

In the MetaDE package, we used MetaDE.Read, MetaDE.merge and MetaDE.filter to read the datasets into R and to extract the common genes across the datasets and filter out unexpressed and non-informative genes. Filtering out unexpressed and non-informative genes resulted in 26 genes among 31 dysregulated genes in PTSD_GSE860_ vs Post-deploy PTSD_GSE63878_ samples and 58 genes among the 132 dysregulated genes in PTSD_GSE860_ vs Pre-deploy PTSD_GSE63878_ vs Post-deploy PTSD_GSE63878_ (Table 1). Significant upregulation of these informative genes was shown in the Fig 3A–3D. Results from the meta-analysis of each gene using each method in MetaDE package were provided in the S6 Table. Volcano plot showing the significant genes in each meta-analysis method was provided in the S3 Fig. Among the different methods in the metaDE package, rOP is demonstrated to be the more generalizable, robust and sensitive statistical framework to detect disease-related markers [40]. Results from the rOP method shows that the genes *OR2B6*, *SOX21*, *MOBP*, *IL15*, *PTPRK* are the most significant DEGs among the PTSD_GSE860_ vs Post-deploy PTSD_GSE63878_ condition and the genes *PPBPP2*, *SEC14L5* are most significant DEGs among the PTSD_GSE860_ vs Pre-deploy PTSD_GSE63878_ vs Post-deploy PTSD_GSE63878_ condition with a low false discovery rate (FDR) and a p-value threshold ≤ 0.05.

**Table 1:** List of genes resulted after filtering out 30% of the non-informative genes using Meta-analysis.

Study	List of genes resulted after filtering
PTSD_GSE860_ vs Post-deploy PTSD_GSE63878_	*DST*, *PCYT1B*, *AMPH*, *LPL*, *CITED1*, *CACNA1G*, *DAZ3///DAZ4///DAZ1///DAZ2*, *DTYMK*, *OR2B6*, *COL5A2*, *SOX21*, *PLK1*, *HBE1*, *ADH1A*, *COL14A1*, *DRD3*, *MOBP*, *NAV3*, *DSCR4*, *ME1*, *SERPINE2*, *OVOL1*, *SND1-IT1*, *IL15*, *PTPRK*, *LRRC32*
PTSD_GSE860_ vs Pre-deploy PTSD_GSE63878_ vs Post-deploy PTSD_GSE63878_	*BLVRA*, *ELANE*, *SLC1A4*, *NCKAP1*,*NTSR2*, *PPBPP2*, *HSPB6*, *DHH*, *CHI3L1*, *HLF*,*CBR1*, *F5*, *TULP3*, *EXOG*, *FANCC*, *SP1*, *CSTF2*, *SEC14L5*, *PTH1R*, *KIAA0930*, *ESPL1*, *MYO16*, *ERCC4*, *GUCY1A3*, *OCM///OCM2*, *SLC1A4*, *TP53TG1*, *NKX3-1*, *EXOG*, *LSAMP*, *VSIG4*, *NR2C2*, *BCL2L2*, *ZFY*, *ERCC4*, *GP5*, *SDC3*, *MAPKAPK5-AS1*, *POLA1*, *RFX3*, *KRT5*, *MSLN*, *RAMP1*, *CCL11*, *HNF1A*, *METTL13*, *DCLRE1A*, *TSR2*, *ZNF271P*, *CDK20*, *P3H4*, *PKI55*, *XCL1*, *CHML*, *MSH6*, *PRELP*, *CD160*, *ZKSCAN5*

### Functional enrichment analysis

Results from GO functional enrichment analysis with a cut-off p-value ≤ 0.05 for the PTSD_GSE860_ vs Post-deploy PTSD_GSE63878_ condition showed i) the molecular functions of the genes were mainly related to cell proliferation, system development, anatomical structure development and multicellular organismal development ii) the cell components involved were mainly in the extracellular matrix iii) the biological processes involved were mainly regulation of locomotion (Fig 3E; S7 Table) whereas results from top three clusters of PTSD_GSE860_ vs Pre-deploy PTSD_GSE63878_ vs Post-deploy PTSD_GSE63878_ condition showed i) the molecular functions of the genes were mainly related to homeostatic process ii) the cell components involved were mainly in the DNA repair iii) the biological processes involved were mainly response to DNA damage stimulus and regulation of DNA metabolic process (Fig 3F; S7 Table).

### Interaction network analysis

**Fig 4:**
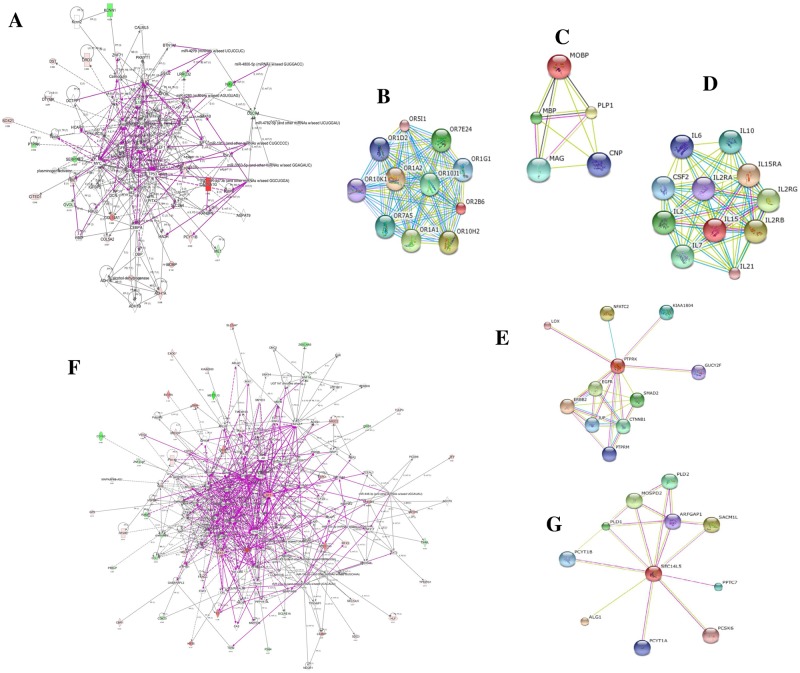
Interaction network analysis of datasets. (A, B, C, D, E) represents interaction network of PTSD_GSE860_ vs Post-deploy PTSD_GSE63878_ condition (F, G) represents interaction network of PTSD_GSE860_ vs Pre- vs Post-deploy PTSD_GSE63878_ condition. In G, L solid lines represent direct interactions and dashed lines represent indirect interactions. In B, C, D, E, G nodes represent the genes and the edges represent their corresponding interactions.

Results from Ingenuity analysis yielded three functional molecular networks for the informative genes in both PTSD_GSE860_ vs Post-deploy PTSD_GSE63878_ and PTSD_GSE860_ vs Pre-deploy PTSD_GSE63878_ vs Post-deploy PTSD_GSE63878_ conditions. Merged forms of the three functional molecular networks for them were presented in the Fig 4A and 4F. Further, among the significant genes predicted by the MetaDE methods, genes *SOX21* and *PPBPP2* failed to form interaction using STRING database in *Homo sapiens* and the interaction networks for the remaining genes were shown in the Fig 4B–4E and 4G.

## Discussion

Genetic factors play a prominent role in the susceptibility of PTSD and the family members of PTSD patients have a higher chance to develop than the other similarly trauma-exposed non-family members [56]. The majority of genes that induce risk in PTSD are also known to confer risk in other psychiatric disorders such as alcohol, drug [10] and nicotine dependence [10, 57]. After exposure to a traumatic event, only a few individuals develop PTSD [58] which is known to be the risk of suicide attempt in the adults with recurrent major depression [59]. A previous population-based study showed that there would be a direct relation between the aggression and post-deployment PTSD [60] and the degree of symptom heterogeneity is known to differ by time point in PTSD [61]. Current understanding of the pathophysiology of PTSD is hampered by the complexity of the human system and lack of understanding regarding how exposures lead to symptoms thereby causing deployment-related psychological injury [62].

Specific genes or biomolecules and biological networks implicated in PTSD are still needed to be identified. Identification of such genes can enlighten our understanding of the factors that may persuade the development, maintenance, or treatment of PTSD. Meta-analysis has been effective in elucidating the gene signatures in several studies [63–65] and has been widely applied to augment statistical power and provide validated conclusions [66]. In the present study, we have analyzed and classified the differential expression of genes in the PTSD microarray datasets using six different microarray meta-analysis methods (Fisher, roP, Stouffer, AW, SR and PR). Here in this study, we elucidated the differentially expressed genes in pre-deployment, post-deployment and PTSD conditions. This is the first meta-analysis to examine the association of informative genes in different conditions of PTSD.

A search for Microarray datasets or profiles for PTSD showed 941 datasets and 194 profiles in GEO database. Previous studies suggested that Peripheral Blood Mononuclear Cells (PBMCs) play an indicator role in a putative mechanism and effective diagnosis of PTSD and they could play a role in the causation and/or exacerbation of PTSD [67]. Based on this criterion, we have selected the datasets GSE860 and GSE63878 for our study. GSE860 dataset had PBMC gene expression profiles with 33 samples and GSE63878 dataset had PBMC gene expression profiles of 96 samples. GSE860 dataset constitutes the samples taken from the survivors of psychological trauma at two time points, in emergency room few hours after trauma and four months later whereas GSE63878 constitute the male subjects participated in either the Marine Resilience Study or the Marine Resiliency Study II.

One of the most important steps in analyzing the microarray datasets is to deal with quality problems which can stem from different sources such as hybridization step [68], and these quality problems can affect the data at different levels of the analysis. Quality analysis can be done before or after data preprocessing step such as normalization. Quality analysis after preprocessing helps to determine the effectiveness of the chosen preprocessing steps and more significantly the suitability and usability of the data for the biological analysis [69]. Therefore, we have performed quality assessment of samples after normalization and excluded four samples which were dissimilar from the other samples in the two datasets and considered 30 samples in GSE860 dataset and 94 samples in the GSE63878 dataset for our analysis.

For multidimensional data sets, in order to determine the differences in the observations and to simplify the analysis and visualization, principal components analysis (PCA) is an important statistical technique. PCA allows recapitulating the way gene responses differ under different conditions thereby providing insights into the underlying factors that are measured in the experiments [70]. To determine the differences in the observation in different conditions in the datasets, we have performed PCA on conditions followed by filtering of probe sets by expression and screening out differentially expressed genes. Results from the PCA and correlation plots showed that the samples in each dataset are clustered properly (S1 and S2 Figs). These clustered samples were then subjected to meta-analysis.

MetaOmics is a software suite for a systematic microarray meta-analysis pipeline containing three integrated R packages namely MetaQC, MetaDE and MetaPath. MetaQC determines the inclusion/exclusion criteria for meta-analysis, MetaDE provides many state-of-the-art genomic meta-analysis methods to detect differentially expressed genes and MetaPath provides inference for detecting enriched pathways associated with outcome [71]. We have determined the most significant differentially expressed and informative genes using the MetDE package. By comparing the expression levels in two datasets, 7 genes were identified as significant among the DEGs, including *OR2B6*, *SOX21*, *MOBP*, *IL15*, *PTPRK*, *PPBPP2* and *SEC14L5*.

*OR2B6* belongs to the olfactory receptors which are reported to initiate neuronal responses triggering the perception of smell [72]. *SOX21* enables the proper gene activation during hippocampal neurogenesis and primes the epigenetic landscape in neural precursors [73, 74]. *MOBP* has been a biomarker in several neuropsychiatric disorders [75]. *IL15* is considered as one of the inflammatory markers for PTSD [76] and is known to be dysregulated in patients suffering from Gulf War Illness [77]. *PTPRK* is known to show a key role in other neurological disorders [78]. *PPBPP2* is a gene belonging to the DNA binding proteins which have a prominent role in PTSD [79]. *SEC14L5* on the other hand is a gene belonging to the subgroup of SEC14-containing proteins located on chromosomes 17 and 16 [80]. *SEC14L5* is found to be significantly altered in the non-child abused PTSD patients [81]. All these genes predicted using our meta-analysis are known to show a direct and indirect relationship to neuro-specific disorders and our finding that they are also important for PTSD will warrant the development of novel therapeutic strategies against PTSD. Future functional studies need to be done elucidating the role of each gene in PTSD.

One of the common procedures to understand the biological data is to check whether the genes drawn in a biological experiment are functionally relevant or not [82] and Gene Ontology is one of the most widespread and integrated approach for functional enrichment analysis. The GO enrichment analysis revealed that DEGs were significantly enriched in biological processes involved in cell proliferation and DNA repair. Among the DEGs, IL15 which is a pro-inflammatory cytokine showed the GO term significantly enriched in cell proliferation at a cut-off p-value ≤ 0.05 which correlates with the recent report that PTSD is coupled with an enhanced spontaneous production of pro-inflammatory cytokines by PBMCs [83]. Besides function enrichment analysis, interaction network was also constructed to visualize the interactions between DEGs and the other genes. Among the seven most significant DEGs predicted using meta-analysis, two genes *IL15* and *SEC14L5* were found to have more interactions than the other genes. Further, STRING database analyses identified a network of 10 genes belonging to olfactory receptor family for *OR2B6* (Fig 4B), 4 genes belonging to the proteolipid and myelin protein for *MOBP* (Fig 4C), 10 genes belonging to cytokines for *IL15* (Fig 4D),10 genes belonging to the signaling pathway proteins and receptors for *PTPRK* (Fig 4E) and 8 genes belonging to lipases and suppressors for *SEC14L5* (Fig 4F). The schematic representation of the overall meta-analysis pipeline used in the study is shown in S4 Fig.

## Conclusion

In conclusion, our present analysis showed that *IL15* and *SEC14L5*, and the other informative genes predicted, can be some of the possible genetic targets for PTSD. The results from the present study will allow a better understanding of the molecular basis of the disease thereby opening up novel strategies for developing effective therapies targeting PTSD.

## Supporting Information

S1 Fig Pearson's Correlation coefficient plot and Principal component analysis of the samples used for our study. (A,B,C) represents the control samples of the GSE860 dataset (D,E,F) represents the PTSD samples of the GSE860 dataset (G,H,I) represents the pre-deployment control samples of the GSE63878 dataset(J,K,L) represents the post-deployment control samples of the GSE63878 dataset (M,N,O) represents the pre-deployment PTSD samples of the GSE63878 dataset (P,Q,R) represents the post-deployment PTSD samples of the GSE63878 dataset.
(TIF)

S2 Fig Correlation and Principal component analysis of the samples after removing the outliers. (A,B,C) represent the control samples of the GSE860 dataset (D,E,F) represents the post-deployment control samples of the GSE63878 dataset (G,H,I) represents the pre-deployment PTSD samples of the GSE63878 dataset.
(TIF)

S3 Fig Volcano plot of genes in different meta-analysis methods. A, B, C, D, E, F represents PTSD_GSE860_ vs Post-deploy PTSD_GSE63878_ whereas G, H, I, J, K, L represents PTSD_GSE860_ vs Pre-deploy PTSD_GSE63878_ vs Post-deploy PTSD_GSE63878_. Red points in the plot represent the significant differentially expressed genes at a p-value < 0.05.
(TIF)

S4 Fig Schematic representation of the meta-analysis pipeline used in the study. (TIF)

S1 Table Characteristics of the individual profiles included in the present study. (DOC)

S2 Table List of Samples in the two datasets considered for our analysis. *PMBC;* Peripheral blood mononuclear cell, *PBL;* Peripheral blood leukocytes, ER; Emergency room.
(DOC)

S3 Table Summary statistics of the datasets used for our study. (XLS)

S4 Table Genes that are commonly dysregulated among PTSD_GSE860_ and Post-deploy PTSD_GSE63878_ samples. (XLS)

S5 Table Genes that are commonly dysregulated among PTSD_GSE860_, Pre-deploy PTSD_GSE63878_ and Post-deploy PTSD_GSE63878_ samples. (XLS)

S6 Table Meta-analysis of up and down regulated genes in (A) PTSD vs Post-deploy (B) PTSD vs Pre-deploy vs Post-deploy conditions. Genes which are most significant using roP method at a P value ≤ 0.05 with a low FDR represented in bold letters.
(DOC)

S7 Table Gene ontology analysis and Functional enrichment analysis using DAVID program. (XLS)
