# Supplementary figures and images for: Prediction of Possible Biomarkers and Novel Pathways Conferring Risk to Post-Traumatic Stress Disorder
Source: PLoS One. 2016 Dec 20;11(12):e0168404. doi: 10.1371/journal.pone.0168404 (PMC5172609; doi:10.1371/journal.pone.0168404)

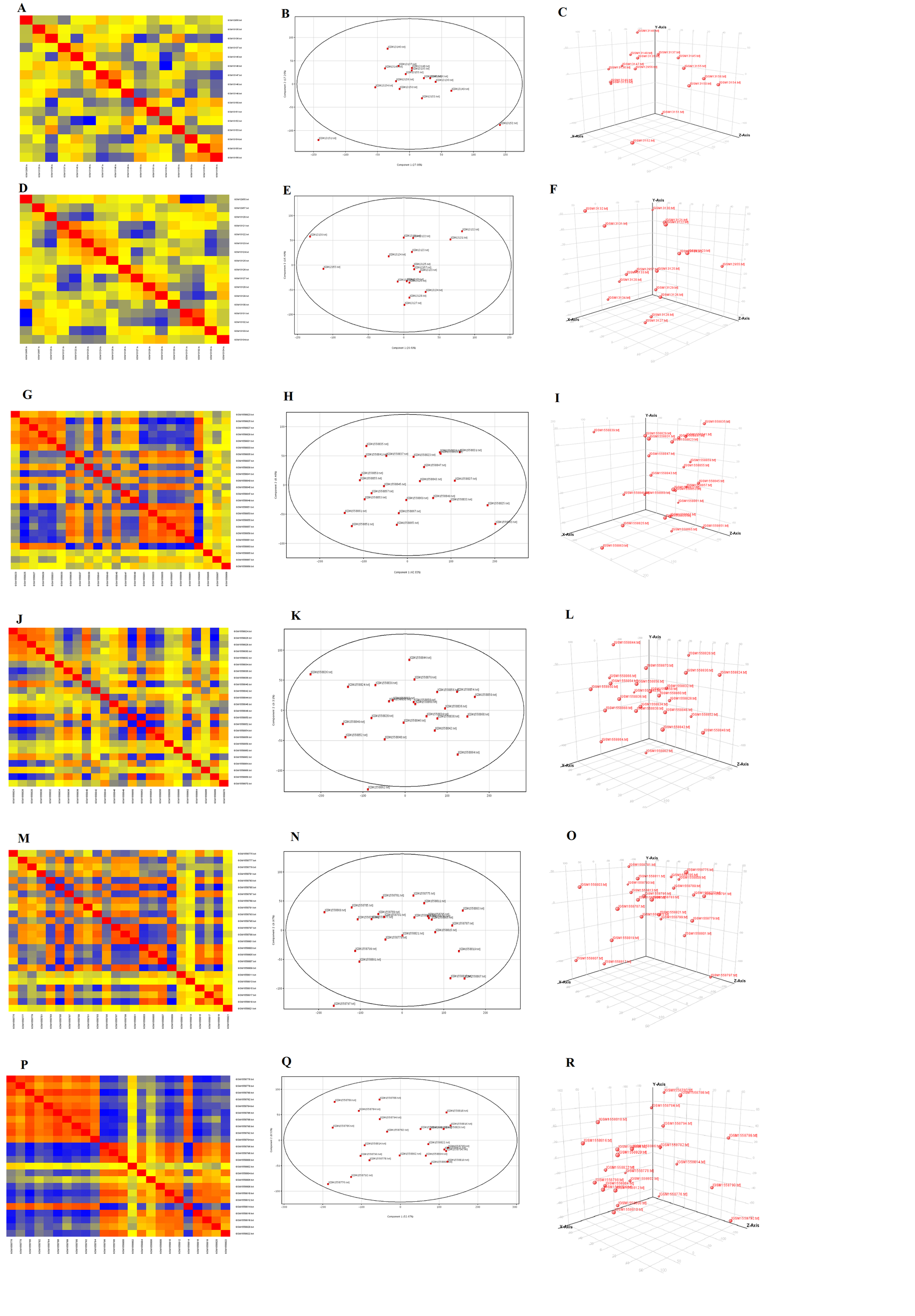

Supplement: S1 Fig — (A,B,C) represents the control samples of the GSE860 dataset (D,E,F) represents the PTSD samples of the GSE860 dataset (G,H,I) represents the pre-deployment control samples of the GSE63878 dataset(J,K,L) represents the post-deployment control samples of the GSE63878 dataset (M,N,O) represents the pre-deployment PTSD samples of the GSE63878 dataset (P,Q,R) represents the post-deployment PTSD samples of the GSE63878 dataset. (TIF) [file pone.0168404.s001.tif]

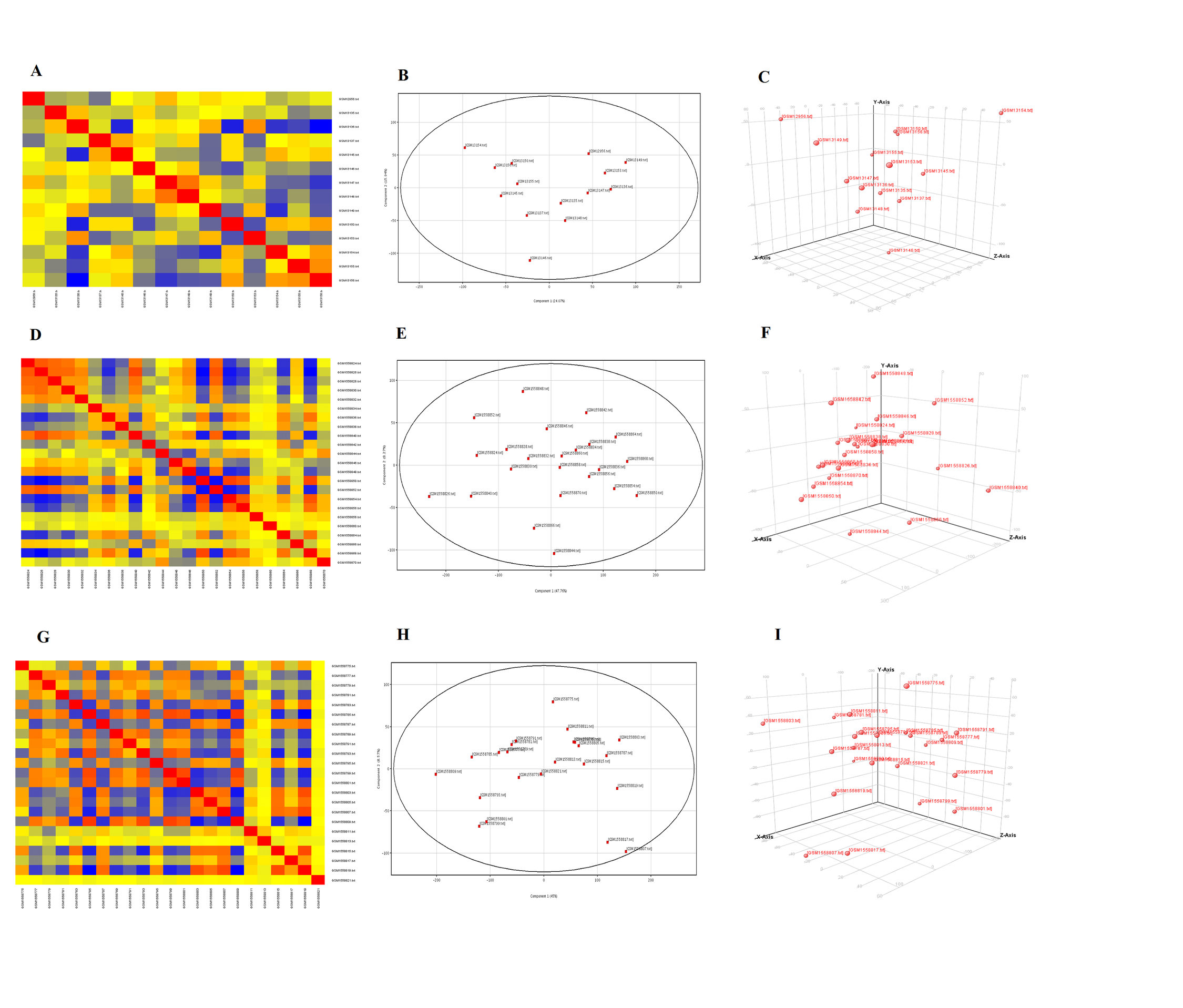

Supplement: S2 Fig — (A,B,C) represent the control samples of the GSE860 dataset (D,E,F) represents the post-deployment control samples of the GSE63878 dataset (G,H,I) represents the pre-deployment PTSD samples of the GSE63878 dataset. (TIF) [file pone.0168404.s002.tif]

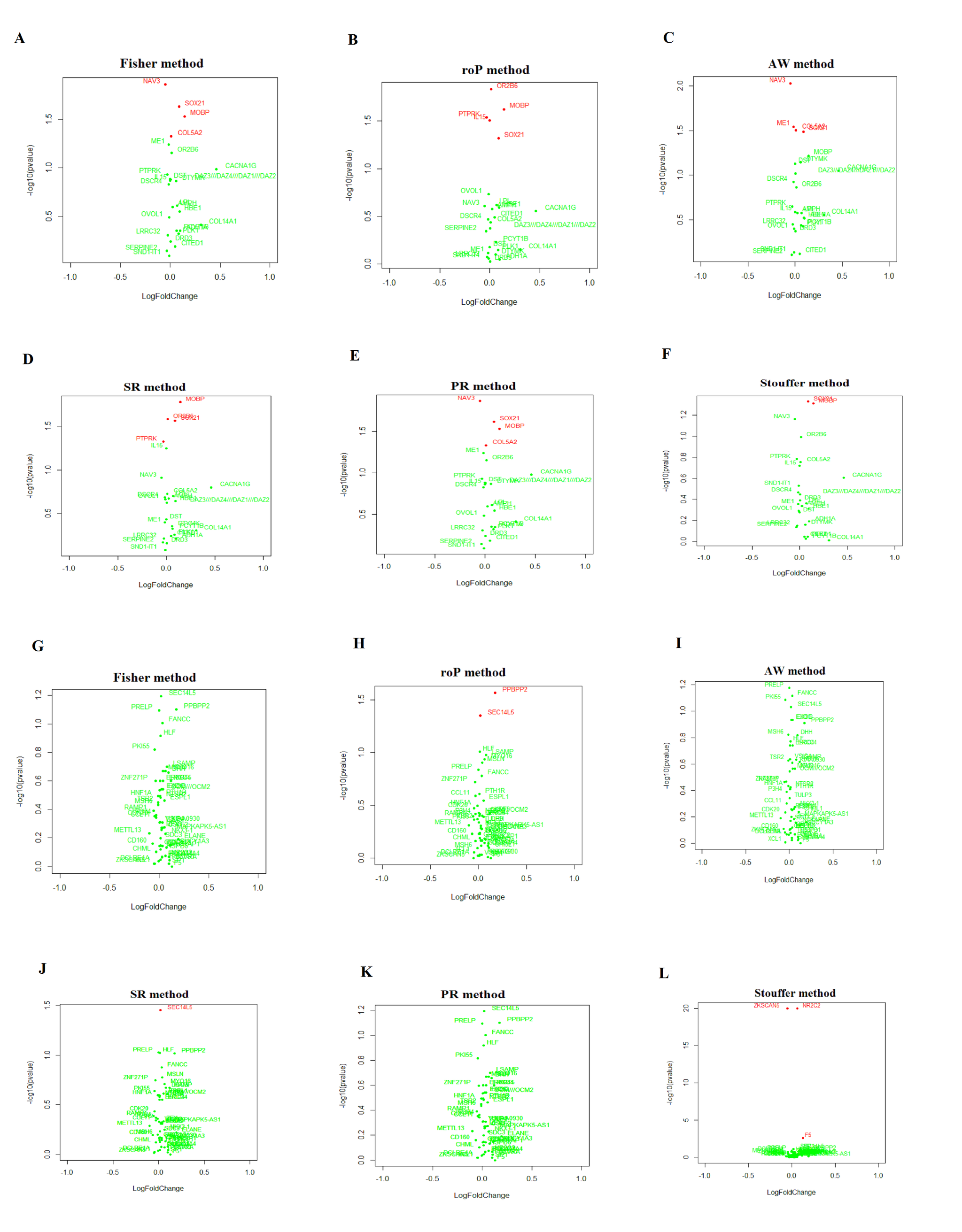

Supplement: S3 Fig — A, B, C, D, E, F represents PTSDGSE860 vs Post-deploy PTSDGSE63878 whereas G, H, I, J, K, L represents PTSDGSE860 vs Pre-deploy PTSDGSE63878 vs Post-deploy PTSDGSE63878. Red points in the plot represent the significant differentially expressed genes at a p-value < 0.05. (TIF) [file pone.0168404.s003.tif]

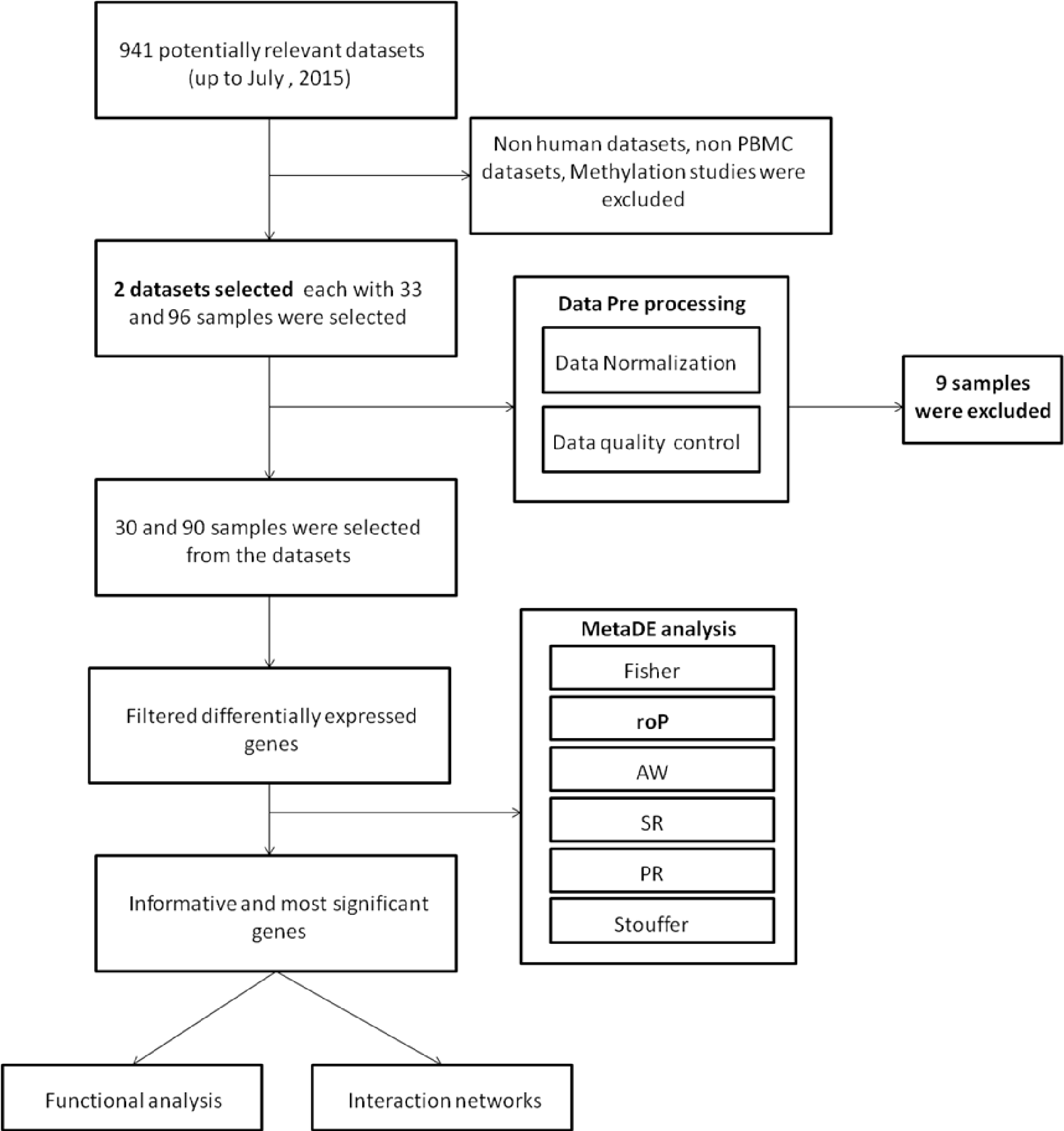

Supplement: S4 Fig — (TIF) [file pone.0168404.s004.tif]
