# Supplementary material for: Prediction of Possible Biomarkers and Novel Pathways Conferring Risk to Post-Traumatic Stress Disorder
Source: PLoS One. 2016 Dec 20;11(12):e0168404. doi: 10.1371/journal.pone.0168404 (PMC5172609; doi:10.1371/journal.pone.0168404)
Supplement: S6 Table — Genes which are most significant using roP method at a P value ≤ 0.05 with a low FDR represented in bold letters. (DOC) [file pone.0168404.s010.doc]

**S6 Table.** Meta-analysis of up and down regulated genes in a) PTSD vs Post-deploy b) PTSD vs Pre-deploy vs Post-deploy conditions. Genes which are most significant using roP method at a P value ≤ 0.05 with a low FDR represented in bold letters.

(**A**)

| **Gene symbol** | **Fisher** | **roP** | **AW** | **SR** | **PR** | **Stouffer** |
| --- | --- | --- | --- | --- | --- | --- |
|  | **p val** | **p val** | **p val** | **p val** | **p val** | **P val** |
| *DST* | 0.1304887125 | 0.6688166708 | 0.0741751426 | 0.3688910940 | 0.1302406351 | 5.286529e-01 |
| *PCYT1B* | 0.4420739271 | 0.5926569090 | 0.3641776234 | 0.4735797569 | 0.4428181593 | 9.404614e-01 |
| *AMPH* | 0.2527908708 | 0.2642024312 | 0.2627139668 | 0.2121061771 | 0.2515504838 | 4.586951e-01 |
| *DAZ3///DAZ4///DAZ1///DAZ2* | 0.1324733317 | 0.4224758125 | 0.0960059539 | 0.2388985363 | 0.1322252543 | 3.557430e-01 |
| *LPL* | 0.2446043165 | 0.2396427685 | 0.2627139668 | 0.1974696105 | 0.2443562391 | 4.321508e-01 |
| *CITED1* | 0.6477300918 | 0.3237410072 | 0.7524187547 | 0.5685934011 | 0.6482262466 | 9.037460e-01 |
| *DTYMK* | 0.1366906475 | 0.8010419251 | 0.0719424460 | 0.4413296949 | 0.1352021831 | 6.938725e-01 |
| ***OR2B6*** | **0.0704539816** | **0.0146365666** | **0.1369387249** | **0.0260481270** | **0.0704539816** | **1.024560e-01** |
| *CACNA1G* | 0.1036963533 | 0.2778466882 | 0.0888117093 | 0.1577772265 | 0.1044405855 | 2.475812e-01 |
| *COL5A2* | 0.0471347060 | 0.3669064748 | 0.0312577524 | 0.1868022823 | 0.0463904738 | 1.768792e-01 |
| ***SOX21*** | **0.0235673530** | **0.0478789382** | **0.0324981394** | **0.0272885140** | **0.0240635078** | **4.688663e-02** |
| *PLK1* | 0.4770528405 | 0.7134706028 | 0.3693872488 | 0.5504837509 | 0.4768047631 | 8.997767e-01 |
| *HBE1* | 0.2818159266 | 0.2570081866 | 0.3011659638 | 0.2272388985 | 0.2828082362 | 4.889606e-01 |
| *ADH1A* | 0.4460431655 | 0.9000248077 | 0.3024063508 | 0.6077896304 | 0.4477797073 | 6.417762e-01 |
| *COL14A1* | 0.3855122798 | 0.7085090548 | 0.2778466882 | 0.4931778715 | 0.3862565120 | 9.712230e-01 |
| *DRD3* | 0.5745472587 | 0.9404614240 | 0.4232200447 | 0.6866782436 | 0.5747953361 | 4.080873e-01 |
| ***MOBP*** | **0.0295212106** | **0.0238154304** | **0.0602828082** | **0.0166211858** | **0.0295212106** | **4.887125e-02** |
| *NAV3* | 0.0138923344 | 0.2470850905 | 0.0094269412 | 0.1223021583 | 0.0136442570 | 6.896552e-02 |
| *DSCR4* | 0.1485983627 | 0.3411064252 | 0.1188290747 | 0.2049119325 | 0.1485983627 | 3.416026e-01 |
| *ME1* | 0.0575539568 | 0.7658149343 | 0.0285289010 | 0.3979161498 | 0.0578020342 | 4.415778e-01 |
| *SERPINE2* | 0.7194244604 | 0.4562143389 | 0.7712726371 | 0.6747705284 | 0.7186802282 | 7.276110e-01 |
| *OVOL1* | 0.3254783759 | 0.1855618953 | 0.3979161498 | 0.2165715703 | 0.3257256264 | 5.100480e-01 |
| *SND1-IT1* | 0.8015380799 | 0.8816670801 | 0.7290994790 | 0.8233688911 | 0.8010419251 | 2.959563e-01 |
| ***IL15*** | **0.1356983379** | **0.0310096750** | **0.2557677996** | **0.0565616472** | **0.1366906475** | **1.900273e-01** |
| ***PTPRK*** | **0.1168444555** | **0.0290250558** | **0.2227735053** | **0.0471347060** | **0.1173406103** | **1.647234e-01** |
| *LRRC32* | 0.4902009427 | 0.8461920119 | 0.3547506822 | 0.6087819400 | 0.4911932523 | 7.035475e-01 |

**(B)**

| **Gene symbol** | **Fisher** | **roP** | **AW** | **SR** | **PR** | **Stouffer** |
| --- | --- | --- | --- | --- | --- | --- |
|  | **p val** | **p val** | **p val** | **p val** | **p val** | **p val** |
| *BLVRA* | 0.9072420635 | 0.7301587302 | 0.931051587 | 0.9025297619 | 0.9072420635 | 1.867560e-01 |
| *ELANE* | 0.6453373016 | 0.5441468254 | 0.694692460 | 0.6021825397 | 0.6455853175 | 8.055556e-01 |
| *SLC1A4* | 0.8583829365 | 0.7646329365 | 0.950396825 | 0.8363095238 | 0.8583829365 | 4.208829e-01 |
| *NCKAP1* | 0.7373511905 | 0.6679067460 | 0.701388889 | 0.7358630952 | 0.7380952381 | 5.128968e-01 |
| *NTSR2* | 0.3293650794 | 0.3829365079 | 0.377232143 | 0.2564484127 | 0.3288690476 | 5.778770e-01 |
| ***PPBPP2*** | **0.0793650794** | **0.0270337302** | **0.123015873** | **0.0959821429** | **0.0788690476** | **1.493056e-01** |
| *HSPB6* | 0.7601686508 | 0.5935019841 | 0.778273810 | 0.7549603175 | 0.7611607143 | 4.499008e-01 |
| *DHH* | 0.2189980159 | 0.4548611111 | 0.152281746 | 0.2447916667 | 0.2189980159 | 4.476687e-01 |
| *CHI3L1* | 0.7127976190 | 0.7998511905 | 0.579365079 | 0.7604166667 | 0.7127976190 | 3.648313e-01 |
| *HLF* | 0.1217757937 | 0.0977182540 | 0.169642857 | 0.0944940476 | 0.1207837302 | 2.065972e-01 |
| *CBR1* | 0.7204861111 | 0.5337301587 | 0.760664683 | 0.7152777778 | 0.7212301587 | 5.327381e-01 |
| *F5* | 0.9999999999 | 0.9995039683 | 1.000000000 | 0.9999999999 | 0.9999999999 | 2.728175e-03 |
| *TULP3* | 0.4913194444 | 0.4583333333 | 0.457589286 | 0.4692460317 | 0.4915674603 | 9.704861e-01 |
| *EXOG* | 0.2869543651 | 0.6827876984 | 0.116815476 | 0.4930555556 | 0.2867063492 | 8.598710e-01 |
| *FANCC* | 0.0984623016 | 0.1656746032 | 0.076388889 | 0.1329365079 | 0.0994543651 | 2.113095e-01 |
| *SP1* | 0.9568452381 | 0.9856150794 | 0.897073413 | 0.9605654762 | 0.9570932540 | 8.730159e-02 |
| *CSTF2* | 0.5374503968 | 0.5252976190 | 0.561507937 | 0.4779265873 | 0.5379464286 | 9.838790e-01 |
| ***SEC14L5*** | **0.0639880952** | **0.0446428571** | **0.092757937** | **0.0352182540** | **0.0639880952** | **8.680556e-02** |
| *PTH1R* | 0.3204365079 | 0.2467757937 | 0.391121032 | 0.2636408730 | 0.3211805556 | 5.835813e-01 |
| *KIAA0930* | 0.4900793651 | 0.9312996032 | 0.246031746 | 0.6832837302 | 0.4903273810 | 6.034226e-01 |
| *ESPL1* | 0.3444940476 | 0.2839781746 | 0.550843254 | 0.2433035714 | 0.3444940476 | 5.778770e-01 |
| *MYO16* | 0.2137896825 | 0.1155753968 | 0.272073413 | 0.1959325397 | 0.2137896825 | 4.119544e-01 |
| *ERCC4* | 0.2512400794 | 0.3990575397 | 0.182291667 | 0.2817460317 | 0.2509920635 | 5.277778e-01 |
| *GUCY1A3* | 0.7061011905 | 0.5411706349 | 0.735615079 | 0.6959325397 | 0.7075892857 | 5.905258e-01 |
| *OCM///OCM2* | 0.2919146825 | 0.3750000000 | 0.286458333 | 0.2519841270 | 0.2914186508 | 5.436508e-01 |
| *SLC1A4* | 0.8583829365 | 0.7646329365 | 0.950396825 | 0.8363095238 | 0.8583829365 | 4.208829e-01 |
| *TP53TG1* | 0.8995535714 | 0.9340277778 | 0.837053571 | 0.9032738095 | 0.8998015873 | 2.333829e-01 |
| *NKX3-1* | 0.5920138889 | 0.7160218254 | 0.531498016 | 0.5654761905 | 0.5920138889 | 8.710317e-01 |
| *EXOG* | 0.2869543651 | 0.6827876984 | 0.116815476 | 0.4930555556 | 0.2867063492 | 8.598710e-01 |
| *LSAMP* | 0.1994047619 | 0.1056547619 | 0.233630952 | 0.2137896825 | 0.1989087302 | 4.191468e-01 |
| *VSIG4* | 0.4898313492 | 0.9521329365 | 0.232142857 | 0.7155257937 | 0.4880952381 | 4.412202e-01 |
| *NR2C2* | 0.8447420635 | 0.6584821429 | 0.860119048 | 0.8549107143 | 0.8449900794 | 1.000000e-20 |
| *BCL2L2* | 0.7167658730 | 0.5905257937 | 0.790674603 | 0.6805555556 | 0.7172619048 | 6.763393e-01 |
| *ZFY* | 0.5339781746 | 0.4055059524 | 0.660218254 | 0.4625496032 | 0.5332341270 | 9.570933e-01 |
| *ERCC4* | 0.2512400794 | 0.3990575397 | 0.182291667 | 0.2817460317 | 0.2509920635 | 5.277778e-01 |
| *GP5* | 0.7155257937 | 0.6460813492 | 0.781001984 | 0.6753472222 | 0.7160218254 | 6.912202e-01 |
| *SDC3* | 0.6356646825 | 0.5798611111 | 0.676339286 | 0.5848214286 | 0.6351686508 | 8.387897e-01 |
| *MAPKAPK5-AS1* | 0.5550595238 | 0.5235615079 | 0.635168651 | 0.4811507937 | 0.5555555556 | 9.918155e-01 |
| *POLA1* | 0.8601190476 | 0.7628968254 | 0.912946429 | 0.8444940476 | 0.8606150794 | 3.948413e-01 |
| *RFX3* | 0.5349702381 | 0.4957837302 | 0.552579365 | 0.4838789683 | 0.5352182540 | 9.947917e-01 |
| *KRT5* | 0.2517361111 | 0.3722718254 | 0.241567460 | 0.2132936508 | 0.2517361111 | 4.598214e-01 |
| *MSLN* | 0.2140376984 | 0.1240079365 | 0.272073413 | 0.1666666667 | 0.2142857143 | 3.807044e-01 |
| *RAMP1* | 0.4069940476 | 0.4017857143 | 0.344990079 | 0.4094742063 | 0.4067460317 | 8.365575e-01 |
| *CCL11* | 0.4541170635 | 0.2569444444 | 0.502480159 | 0.4551091270 | 0.4541170635 | 9.935516e-01 |
| *HNF1A* | 0.3211805556 | 0.3204365079 | 0.370287698 | 0.2542162698 | 0.3214285714 | 5.701885e-01 |
| *METTL13* | 0.5865575397 | 0.4905753968 | 0.647817460 | 0.5166170635 | 0.5870535714 | 3.395337e-01 |
| *DCLRE1A* | 0.9206349206 | 0.9503968254 | 0.862351190 | 0.9218750000 | 0.9206349206 | 1.937004e-01 |
| *TSR2* | 0.3546626984 | 0.4288194444 | 0.236111111 | 0.4392361111 | 0.3546626984 | 8.841766e-01 |
| *ZNF271P* | 0.2519841270 | 0.1912202381 | 0.342261905 | 0.1790674603 | 0.2522321429 | 4.293155e-01 |
| *CDK20* | 0.4437003968 | 0.3365575397 | 0.585565476 | 0.3690476190 | 0.4441964286 | 7.822421e-01 |
| *P3H4* | 0.4352678571 | 0.3819444444 | 0.409474206 | 0.4246031746 | 0.4350198413 | 8.799603e-01 |
| *PKI55* | 0.1512896825 | 0.4320436508 | 0.081845238 | 0.2296626984 | 0.1522817460 | 3.541667e-01 |
| *XCL1* | 0.9536210317 | 0.8891369048 | 0.985119048 | 0.9461805556 | 0.9533730159 | 2.065972e-01 |
| *CHML* | 0.7929067460 | 0.6887400794 | 0.857886905 | 0.7611607143 | 0.7929067460 | 5.548115e-01 |
| *MSH6* | 0.3692956349 | 0.8082837302 | 0.150545635 | 0.6324404762 | 0.3680555556 | 6.168155e-01 |
| *PRELP* | 0.0803571429 | 0.1450892857 | 0.066468254 | 0.0942460317 | 0.0806051587 | 1.470734e-01 |
| *CD160* | 0.6907242063 | 0.5927579365 | 0.782738095 | 0.6401289683 | 0.6907242063 | 7.527282e-01 |
| *ZKSCAN5* | 0.9523809524 | 0.9999999999 | 0.832093254 | 0.9650297619 | 0.9528769841 | 1.000000e-20 |
